# Dicyclo­hexyl­ammonium bis­(chloro­difluoro­acetato-κ*O*)cyclo­pentyl­diphenyl­stannate(IV)

**DOI:** 10.1107/S1600536808010775

**Published:** 2008-04-23

**Authors:** Yin Yin Teo, Kong Mun Lo, Seik Weng Ng

**Affiliations:** a Department of Chemistry, University of Malaya, 50603 Kuala Lumpur, Malaysia

## Abstract

The five-coordinate Sn atom in the title mixed organyl stannate compound, (C_12_H_24_N)[Sn(C_5_H_9_)(C_6_H_5_)_2_(C_2_ClF_2_O_2_)], is in a *trans*-C_3_SnO_2_ trigonal–bipyramidal coordination environment. The NH_2_ groups of the cations act as hydrogen-bond donors to two symmetry-related anions, resulting in the formation of linear chains. One of the phenyl rings is disordered over two sites with equal occupancies.

## Related literature

For details of the crystal structure of dicyclo­hexyl­ammonium bis­(chloro­difluoro­acetato)cyclo­hexyl­diphenyl­stannate(IV), see Teo *et al.* (2008 ▶). For a review of the structural chemistry of organotin carboxyl­ates, see: Tiekink (1991 ▶, 1994 ▶).
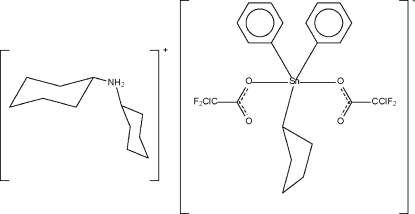


## Experimental

### 

#### Crystal data

(C_12_H_24_N)[Sn(C_5_H_9_)(C_6_H_5_)_2_(C_2_ClF_2_O_2_)]
*M* *_r_* = 783.27
Monoclinic, 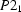

*a* = 8.8610 (2) Å
*b* = 19.3132 (3) Å
*c* = 10.6823 (2) Å
β = 109.385 (1)°
*V* = 1724.47 (6) Å^3^
*Z* = 2
Mo *K*α radiation
μ = 0.95 mm^−1^
*T* = 100 (2) K
0.30 × 0.20 × 0.15 mm

#### Data collection

Bruker SMART APEXII diffractometer
Absorption correction: multi-scan (*SADABS*; Sheldrick, 1996 ▶) *T*_min_ = 0.679, *T*_max_ = 0.870
18017 measured reflections
7831 independent reflections
6637 reflections with *I* > 2σ(*I*)
*R*_int_ = 0.038

#### Refinement

*R*[*F* ^2^ > 2σ(*F* ^2^)] = 0.050
*wR*(*F* ^2^) = 0.132
*S* = 1.04
7831 reflections
421 parameters
41 restraints
H-atom parameters constrained
Δρ_max_ = 1.11 e Å^−3^
Δρ_min_ = −0.71 e Å^−3^
Absolute structure: Flack (1983 ▶), 3766 Friedel pairs
Flack parameter: −0.03 (3)

### 

Data collection: *APEX2* (Bruker, 2007 ▶); cell refinement: *SAINT* (Bruker, 2007 ▶); data reduction: *SAINT*; program(s) used to solve structure: *SHELXS97* (Sheldrick, 2008 ▶); program(s) used to refine structure: *SHELXL97* (Sheldrick, 2008 ▶); molecular graphics: *X-SEED* (Barbour, 2001 ▶); software used to prepare material for publication: *publCIF* (Westrip, 2008 ▶).

## Supplementary Material

Crystal structure: contains datablocks global, I. DOI: 10.1107/S1600536808010775/lh2611sup1.cif

Structure factors: contains datablocks I. DOI: 10.1107/S1600536808010775/lh2611Isup2.hkl

Additional supplementary materials:  crystallographic information; 3D view; checkCIF report

## Figures and Tables

**Table:** 

Sn1—C1	2.147 (5)
Sn1—C7	2.136 (6)
Sn1—C13	2.117 (6)
Sn1—O1	2.287 (4)
Sn1—O3	2.249 (4)

**Table:** 

C1—Sn1—C7	119.2 (2)
C1—Sn1—C13	121.7 (2)
C1—Sn1—O1	91.3 (2)
C1—Sn1—O3	90.9 (2)
C7—Sn1—C13	118.9 (2)
C7—Sn1—O1	90.6 (2)
C7—Sn1—O3	87.8 (2)
C13—Sn1—O1	82.8 (3)
C13—Sn1—O3	96.5 (3)
O1—Sn1—O3	177.7 (2)

**Table 2:** Hydrogen-bond geometry (Å, °)

*D*—H⋯*A*	*D*—H	H⋯*A*	*D*⋯*A*	*D*—H⋯*A*
N1—H1*N*1⋯O2	0.88	1.88	2.758 (6)	173
N1—H1*N*2⋯O4^i^	0.88	1.93	2.804 (6)	169

Symmetry code: (i) 
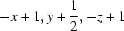
.

**Fig. 1.**
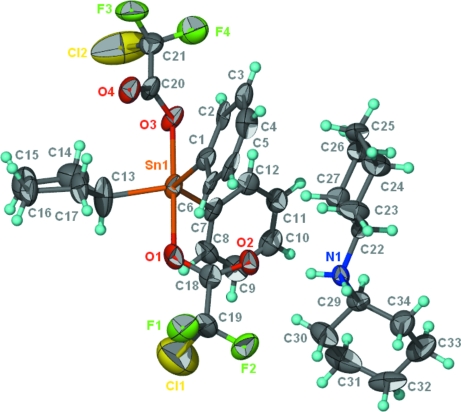
50% Probability thermal ellipsoid plot of the two independent formula units of [(C6H11)2NH2)] [Sn(C5H9)(C6H5)2(O2CClF2)2]. Hydrogen atoms are drawn as spheres of arbitrary radius. The disorder is not shown.

## supplementary crystallographic information

### Comment

The preceding study reports the crystal structure of dicyclohexylammonium bis(chlorodifluroacetato)cyclohexyldiphenylstannate (Teo *et al.*, 2008).

Replacing the cyclohexyl ligand on tin by the cyclopentyl ligand furnishes a similar stannate compound(I), (Fig. 1). The cation engages in hydrogen bonding to the anion to give rise to a chain structure.

### Experimental

Cyclopentyldiphenyltin hydroxide (0.36 g, 1 mmol) was added to an ethanol solution (50 ml) of dicyclohexylamine (0.20 ml, 2 mmol) and chlorodifluoroacetic acid (0.1 ml, 2 mmol). The solution was heated to dissolve the reactants completely; the filtered solution yielded the salt in 70% yield.

### Refinement

Carbon-bound H-atoms were placed in calculated positions (C—H 0.95 to 0.99 Å) and were included in the refinement in the riding model approximation, with *U*(H) set to 1.2*U*_eq_(C). The ammonium H atoms (N–H 0.88 Å) were similarly treated.

For the cyclopentyl ligand, the 1,2-relatd C–C distances were restrained to 1.54±0.01 Å and the 1,3-related one to 2.51 + 0.01 Å. One of the aromatic rings is disordered over two sites; the occupancies could not be refined, and was assumed to be 1:1. For this ring, the 1,2-related distances were restrained to 1.39±0.01 Å and the 1,4-related ones to 2.78±0.01 Å. The temperature factors of the primed atoms were set to those of the unprimed ones; the six atoms of each ring were restrained to be approximately flat.

There is minor disorder in the chlorodifluoromethyl groups; the C–Cl distance was restrained to 1.75±0.01 Å and the C–F distances to 1.35±0.01 Å. The final difference Fourier map had a large peak in the vicinity of Sn1.

### Crystal data

**Table:**

(C_12_H_24_N)[Sn(C_5_H_9_)(C_6_H_5_)_2_(C_2_ClF_2_O_2_)]	*F*_000_ = 800
*M**_r_* = 783.27	*D*_x_ = 1.508 Mg m^−^^3^
Monoclinic, *P*2_1_	Mo *K*α radiation λ = 0.71073 Å
Hall symbol: P 2yb	Cell parameters from 4999 reflections
*a* = 8.8610 (2) Å	θ = 2.3–24.4º
*b* = 19.3132 (3) Å	µ = 0.95 mm^−^^1^
*c* = 10.6823 (2) Å	*T* = 100 (2) K
β = 109.385 (1)º	Irregular block, colorless
*V* = 1724.47 (6) Å^3^	0.30 × 0.20 × 0.15 mm
*Z* = 2	

### Data collection

**Table:**

Bruker SMART APEX diffractometer	7831 independent reflections
Radiation source: fine-focus sealed tube	6637 reflections with *I* > 2σ(*I*)
Monochromator: graphite	*R*_int_ = 0.038
*T* = 100(2) K	θ_max_ = 27.5º
ω scans	θ_min_ = 2.0º
Absorption correction: multi-scan(SADABS; Sheldrick, 1996)	*h* = −11→10
*T*_min_ = 0.679, *T*_max_ = 0.870	*k* = −25→25
18017 measured reflections	*l* = −13→13

### Refinement

**Table:**

Refinement on *F*^2^	Hydrogen site location: inferred from neighbouring sites
Least-squares matrix: full	H-atom parameters constrained
*R*[*F*^2^ > 2σ(*F*^2^)] = 0.050	*w* = 1/[σ^2^(*F*_o_^2^) + (0.0717*P*)^2^ + 1.4264*P*] where *P* = (*F*_o_^2^ + 2*F*_c_^2^)/3
*wR*(*F*^2^) = 0.132	(Δ/σ)_max_ = 0.001
*S* = 1.04	Δρ_max_ = 1.11 e Å^−^^3^
7831 reflections	Δρ_min_ = −0.71 e Å^−^^3^
421 parameters	Extinction correction: none
41 restraints	Absolute structure: Flack (1983), 3766 Friedel pairs
Primary atom site location: structure-invariant direct methods	Flack parameter: −0.03 (3)
Secondary atom site location: difference Fourier map	

### Fractional atomic coordinates and isotropic or equivalent isotropic displacement parameters (Å^2^)

**Table:**

	*x*	*y*	*z*	*U*_iso_*/*U*_eq_	Occ. (<1)
Sn1	0.41483 (4)	0.50001 (7)	0.27785 (3)	0.03273 (11)	
Cl1	0.5652 (5)	0.7213 (2)	0.0894 (3)	0.1213 (13)	
Cl2	0.1779 (5)	0.25792 (18)	0.2051 (3)	0.1310 (16)	
F1	0.7544 (5)	0.7106 (2)	0.3078 (5)	0.0684 (13)	
F2	0.5523 (5)	0.7777 (2)	0.2839 (5)	0.0622 (12)	
F3	0.3331 (6)	0.2399 (2)	0.4277 (5)	0.0721 (14)	
F4	0.1352 (6)	0.3071 (3)	0.4035 (6)	0.0776 (15)	
O1	0.5515 (5)	0.5970 (2)	0.2551 (5)	0.0389 (10)	
O2	0.4078 (5)	0.6683 (2)	0.3391 (5)	0.0372 (10)	
O3	0.2746 (5)	0.4049 (2)	0.2913 (4)	0.0366 (9)	
O4	0.4990 (6)	0.3475 (2)	0.4043 (5)	0.0419 (10)	
N1	0.2491 (6)	0.7638 (2)	0.4417 (4)	0.0264 (9)	
H1N1	0.2976	0.7356	0.4026	0.032*	
H1N2	0.3196	0.7947	0.4861	0.032*	
C1	0.5036 (5)	0.5092 (2)	0.4901 (5)	0.0335 (12)	
C2	0.445 (4)	0.4703 (16)	0.574 (4)	0.034 (3)	0.50
H2	0.3604	0.4389	0.5336	0.040*	0.50
C3	0.502 (6)	0.474 (2)	0.711 (4)	0.040 (5)	0.50
H3	0.4581	0.4462	0.7645	0.047*	0.50
C4	0.625 (6)	0.520 (3)	0.7657 (10)	0.0475 (18)	0.50
H4	0.6666	0.5237	0.8597	0.057*	0.50
C5	0.693 (5)	0.561 (2)	0.692 (4)	0.037 (4)	0.50
H5	0.7779	0.5926	0.7332	0.045*	0.50
C6	0.6282 (18)	0.5547 (12)	0.555 (4)	0.033 (3)	0.50
H6	0.6714	0.5826	0.5018	0.039*	0.50
C2'	0.420 (4)	0.4798 (14)	0.567 (4)	0.034 (3)	0.50
H2'	0.3212	0.4565	0.5261	0.040*	0.50
C3'	0.484 (6)	0.486 (2)	0.703 (4)	0.040 (5)	0.50
H3'	0.4279	0.4657	0.7564	0.047*	0.50
C4'	0.627 (6)	0.519 (3)	0.7660 (10)	0.0475 (18)	0.50
H4'	0.6697	0.5224	0.8599	0.057*	0.50
C5'	0.707 (5)	0.548 (2)	0.686 (4)	0.037 (4)	0.50
H5'	0.8061	0.5706	0.7278	0.045*	0.50
C6'	0.6483 (16)	0.5438 (12)	0.549 (4)	0.033 (3)	0.50
H6'	0.7046	0.5640	0.4959	0.039*	0.50
C7	0.1931 (7)	0.5482 (3)	0.1691 (6)	0.0315 (12)	
C8	0.1848 (8)	0.5921 (3)	0.0642 (6)	0.0373 (13)	
H8	0.2746	0.5977	0.0355	0.045*	
C9	0.0415 (8)	0.6283 (4)	0.0009 (7)	0.0447 (15)	
H9	0.0348	0.6580	−0.0718	0.054*	
C10	−0.0874 (8)	0.6213 (3)	0.0426 (6)	0.0409 (14)	
H10	−0.1823	0.6469	0.0000	0.049*	
C11	−0.0815 (7)	0.5781 (3)	0.1447 (7)	0.0373 (13)	
H11	−0.1720	0.5731	0.1728	0.045*	
C12	0.0584 (7)	0.5410 (3)	0.2081 (6)	0.0340 (12)	
H12	0.0619	0.5105	0.2788	0.041*	
C13	0.5521 (7)	0.4533 (4)	0.1721 (7)	0.056 (2)	
H13	0.5677	0.4912	0.1139	0.068*	
C14	0.4920 (7)	0.3925 (4)	0.0773 (8)	0.062 (2)	
H14A	0.4110	0.4081	−0.0064	0.075*	
H14B	0.4437	0.3565	0.1182	0.075*	
C15	0.6441 (9)	0.3636 (4)	0.0507 (7)	0.074 (3)	
H15A	0.6432	0.3123	0.0501	0.089*	
H15B	0.6480	0.3802	−0.0358	0.089*	
C16	0.7884 (7)	0.3909 (4)	0.1650 (7)	0.0494 (17)	
H16A	0.8564	0.4204	0.1297	0.059*	
H16B	0.8539	0.3519	0.2142	0.059*	
C17	0.7225 (7)	0.4334 (4)	0.2575 (6)	0.0433 (15)	
H17A	0.7215	0.4054	0.3348	0.052*	
H17B	0.7883	0.4753	0.2898	0.052*	
C18	0.5096 (7)	0.6545 (3)	0.2852 (6)	0.0358 (13)	
C19	0.5981 (7)	0.7165 (3)	0.2514 (6)	0.0458 (16)	
C20	0.3529 (8)	0.3533 (3)	0.3501 (7)	0.0358 (14)	
C21	0.2525 (7)	0.2913 (3)	0.3561 (5)	0.0415 (14)	
C22	0.1928 (7)	0.7229 (3)	0.5380 (6)	0.0306 (12)	
H22	0.1109	0.7509	0.5611	0.037*	
C23	0.3314 (9)	0.7076 (3)	0.6660 (6)	0.0437 (15)	
H23A	0.4127	0.6786	0.6458	0.052*	
H23B	0.3830	0.7514	0.7063	0.052*	
C24	0.2667 (10)	0.6697 (4)	0.7623 (7)	0.0514 (18)	
H24A	0.1915	0.7003	0.7870	0.062*	
H24B	0.3563	0.6585	0.8441	0.062*	
C25	0.1806 (9)	0.6031 (3)	0.7024 (7)	0.0423 (15)	
H25A	0.2590	0.5698	0.6893	0.051*	
H25B	0.1325	0.5820	0.7648	0.051*	
C26	0.0522 (8)	0.6162 (3)	0.5720 (7)	0.0433 (15)	
H26A	−0.0349	0.6433	0.5874	0.052*	
H26B	0.0069	0.5714	0.5318	0.052*	
C27	0.1156 (8)	0.6560 (3)	0.4746 (6)	0.0361 (13)	
H27A	0.1950	0.6272	0.4514	0.043*	
H27B	0.0263	0.6662	0.3920	0.043*	
C29	0.1158 (7)	0.8005 (3)	0.3367 (6)	0.0317 (12)	
H29	0.0177	0.7711	0.3160	0.038*	
C30	0.1550 (8)	0.8094 (4)	0.2104 (7)	0.0463 (16)	
H30A	0.1732	0.7635	0.1766	0.056*	
H30B	0.2541	0.8370	0.2286	0.056*	
C31	0.0151 (9)	0.8464 (5)	0.1056 (8)	0.061 (2)	
H31A	0.0441	0.8545	0.0249	0.074*	
H31B	−0.0802	0.8160	0.0809	0.074*	
C32	−0.0264 (9)	0.9144 (4)	0.1539 (8)	0.057 (2)	
H32A	−0.1222	0.9340	0.0862	0.069*	
H32B	0.0632	0.9473	0.1667	0.069*	
C33	−0.0591 (9)	0.9056 (4)	0.2842 (8)	0.057 (2)	
H33A	−0.1579	0.8780	0.2686	0.069*	
H33B	−0.0765	0.9517	0.3178	0.069*	
C34	0.0797 (9)	0.8696 (4)	0.3879 (7)	0.0471 (16)	
H34A	0.1758	0.8994	0.4106	0.057*	
H34B	0.0525	0.8622	0.4696	0.057*	

### Atomic displacement parameters (Å^2^)

**Table:**

	*U*^11^	*U*^22^	*U*^33^	*U*^12^	*U*^13^	*U*^23^
Sn1	0.01949 (15)	0.02969 (16)	0.0492 (2)	0.0012 (2)	0.01167 (12)	−0.0076 (2)
Cl1	0.153 (4)	0.124 (3)	0.103 (2)	−0.027 (2)	0.065 (2)	−0.0111 (19)
Cl2	0.164 (3)	0.085 (2)	0.0910 (19)	−0.061 (2)	−0.029 (2)	0.0079 (15)
F1	0.031 (2)	0.050 (2)	0.128 (4)	−0.0105 (18)	0.031 (2)	0.006 (2)
F2	0.045 (3)	0.035 (2)	0.115 (4)	−0.0008 (18)	0.037 (3)	0.002 (2)
F3	0.062 (3)	0.057 (3)	0.110 (4)	0.019 (2)	0.045 (3)	0.048 (3)
F4	0.049 (3)	0.063 (3)	0.134 (5)	0.010 (2)	0.049 (3)	0.024 (3)
O1	0.021 (2)	0.045 (2)	0.058 (3)	−0.0021 (18)	0.022 (2)	−0.004 (2)
O2	0.027 (2)	0.040 (2)	0.050 (3)	−0.0052 (19)	0.020 (2)	−0.010 (2)
O3	0.024 (2)	0.0246 (19)	0.053 (3)	0.0051 (16)	0.0032 (18)	−0.0040 (17)
O4	0.031 (2)	0.039 (2)	0.054 (3)	0.0095 (19)	0.013 (2)	−0.0006 (19)
N1	0.021 (2)	0.029 (2)	0.031 (2)	−0.0031 (18)	0.0116 (19)	−0.0055 (17)
C1	0.020 (2)	0.027 (3)	0.052 (3)	0.006 (2)	0.011 (2)	0.001 (2)
C2	0.011 (8)	0.037 (7)	0.049 (5)	0.011 (5)	0.007 (6)	−0.012 (4)
C3	0.029 (9)	0.038 (14)	0.054 (5)	0.010 (8)	0.016 (5)	−0.005 (5)
C4	0.043 (4)	0.053 (5)	0.046 (3)	0.007 (3)	0.013 (3)	−0.012 (3)
C5	0.029 (6)	0.023 (13)	0.053 (5)	0.008 (8)	0.003 (5)	−0.018 (7)
C6	0.024 (4)	0.019 (6)	0.054 (5)	0.008 (5)	0.012 (5)	−0.009 (6)
C2'	0.011 (8)	0.037 (7)	0.049 (5)	0.011 (5)	0.007 (6)	−0.012 (4)
C3'	0.029 (9)	0.038 (14)	0.054 (5)	0.010 (8)	0.016 (5)	−0.005 (5)
C4'	0.043 (4)	0.053 (5)	0.046 (3)	0.007 (3)	0.013 (3)	−0.012 (3)
C5'	0.029 (6)	0.023 (13)	0.053 (5)	0.008 (8)	0.003 (5)	−0.018 (7)
C6'	0.024 (4)	0.019 (6)	0.054 (5)	0.008 (5)	0.012 (5)	−0.009 (6)
C7	0.028 (3)	0.022 (2)	0.042 (3)	0.005 (2)	0.009 (2)	−0.007 (2)
C8	0.032 (3)	0.047 (3)	0.036 (3)	−0.006 (3)	0.016 (3)	−0.001 (2)
C9	0.038 (4)	0.053 (4)	0.040 (4)	0.002 (3)	0.010 (3)	0.004 (3)
C10	0.030 (3)	0.039 (3)	0.046 (4)	0.006 (3)	0.003 (3)	−0.004 (3)
C11	0.023 (3)	0.038 (3)	0.053 (4)	−0.001 (2)	0.014 (3)	−0.004 (3)
C12	0.024 (3)	0.033 (3)	0.043 (3)	−0.006 (2)	0.010 (2)	−0.006 (2)
C13	0.028 (3)	0.084 (6)	0.050 (4)	0.015 (4)	0.004 (3)	−0.030 (4)
C14	0.061 (5)	0.066 (5)	0.059 (5)	−0.006 (4)	0.019 (4)	0.005 (4)
C15	0.098 (8)	0.067 (5)	0.070 (6)	0.022 (5)	0.045 (5)	−0.003 (4)
C16	0.039 (4)	0.049 (4)	0.068 (5)	0.013 (3)	0.027 (3)	−0.008 (3)
C17	0.026 (3)	0.059 (4)	0.047 (4)	0.014 (3)	0.015 (3)	−0.004 (3)
C18	0.022 (3)	0.037 (3)	0.046 (3)	−0.005 (2)	0.009 (2)	−0.008 (3)
C19	0.031 (3)	0.049 (4)	0.066 (5)	−0.001 (3)	0.027 (3)	0.005 (3)
C20	0.032 (4)	0.026 (3)	0.050 (4)	0.008 (2)	0.014 (3)	−0.003 (2)
C21	0.035 (3)	0.037 (3)	0.054 (4)	0.005 (3)	0.016 (3)	0.010 (3)
C22	0.032 (3)	0.021 (2)	0.045 (3)	0.001 (2)	0.022 (3)	−0.001 (2)
C23	0.052 (4)	0.042 (3)	0.034 (3)	−0.021 (3)	0.011 (3)	0.000 (3)
C24	0.065 (5)	0.047 (4)	0.047 (4)	−0.019 (3)	0.026 (4)	−0.004 (3)
C25	0.058 (4)	0.025 (3)	0.055 (4)	−0.007 (3)	0.033 (3)	0.002 (3)
C26	0.040 (4)	0.032 (3)	0.064 (4)	−0.009 (3)	0.025 (3)	−0.003 (3)
C27	0.031 (3)	0.030 (3)	0.047 (3)	−0.009 (2)	0.013 (3)	−0.005 (3)
C29	0.023 (3)	0.031 (3)	0.039 (3)	−0.006 (2)	0.008 (2)	0.001 (2)
C30	0.031 (3)	0.066 (5)	0.043 (4)	−0.005 (3)	0.012 (3)	0.008 (3)
C31	0.034 (4)	0.093 (6)	0.050 (4)	−0.012 (4)	0.004 (3)	0.025 (4)
C32	0.034 (4)	0.050 (4)	0.073 (5)	−0.010 (3)	−0.003 (3)	0.030 (4)
C33	0.046 (5)	0.043 (4)	0.075 (5)	0.011 (3)	0.009 (4)	0.011 (3)
C34	0.041 (4)	0.043 (4)	0.055 (4)	0.008 (3)	0.014 (3)	0.003 (3)

### Geometric parameters (Å, °)

**Table:**

Sn1—C1	2.147 (5)	C12—H12	0.9500
Sn1—C7	2.136 (6)	C13—C14	1.527 (7)
Sn1—C13	2.117 (6)	C13—C17	1.530 (7)
Sn1—O1	2.287 (4)	C13—H13	1.0000
Sn1—O3	2.249 (4)	C14—C15	1.569 (7)
Cl1—C19	1.660 (6)	C14—H14A	0.9900
Cl2—C21	1.657 (6)	C14—H14B	0.9900
F1—C19	1.319 (6)	C15—C16	1.539 (7)
F2—C19	1.331 (6)	C15—H15A	0.9900
F3—C21	1.311 (6)	C15—H15B	0.9900
F4—C21	1.334 (6)	C16—C17	1.541 (6)
O1—C18	1.246 (7)	C16—H16A	0.9900
O2—C18	1.249 (7)	C16—H16B	0.9900
O3—C20	1.257 (7)	C17—H17A	0.9900
O4—C20	1.235 (8)	C17—H17B	0.9900
N1—C29	1.508 (7)	C18—C19	1.540 (9)
N1—C22	1.508 (7)	C20—C21	1.506 (9)
N1—H1N1	0.8800	C22—C27	1.512 (8)
N1—H1N2	0.8800	C22—C23	1.534 (9)
C1—C2	1.391 (9)	C22—H22	1.0000
C1—C2'	1.394 (9)	C23—C24	1.522 (9)
C1—C6'	1.396 (9)	C23—H23A	0.9900
C1—C6	1.401 (9)	C23—H23B	0.9900
C2—C3	1.387 (9)	C24—C25	1.524 (9)
C2—H2	0.9500	C24—H24A	0.9900
C3—C4	1.383 (9)	C24—H24B	0.9900
C3—H3	0.9500	C25—C26	1.499 (10)
C4—C5	1.388 (9)	C25—H25A	0.9900
C4—H4	0.9500	C25—H25B	0.9900
C5—C6	1.393 (9)	C26—C27	1.542 (9)
C5—H5	0.9500	C26—H26A	0.9900
C6—H6	0.9500	C26—H26B	0.9900
C2'—C3'	1.386 (9)	C27—H27A	0.9900
C2'—H2'	0.9500	C27—H27B	0.9900
C3'—C4'	1.382 (9)	C29—C30	1.510 (8)
C3'—H3'	0.9500	C29—C34	1.516 (9)
C4'—C5'	1.387 (9)	C29—H29	1.0000
C4'—H4'	0.9500	C30—C31	1.543 (10)
C5'—C6'	1.393 (9)	C30—H30A	0.9900
C5'—H5'	0.9500	C30—H30B	0.9900
C6'—H6'	0.9500	C31—C32	1.499 (12)
C7—C8	1.388 (9)	C31—H31A	0.9900
C7—C12	1.396 (8)	C31—H31B	0.9900
C8—C9	1.410 (9)	C32—C33	1.523 (11)
C8—H8	0.9500	C32—H32A	0.9900
C9—C10	1.365 (10)	C32—H32B	0.9900
C9—H9	0.9500	C33—C34	1.523 (10)
C10—C11	1.361 (9)	C33—H33A	0.9900
C10—H10	0.9500	C33—H33B	0.9900
C11—C12	1.396 (9)	C34—H34A	0.9900
C11—H11	0.9500	C34—H34B	0.9900
			
C1—Sn1—C7	119.2 (2)	C13—C17—C16	105.1 (4)
C1—Sn1—C13	121.7 (2)	C13—C17—H17A	110.7
C1—Sn1—O1	91.3 (2)	C16—C17—H17A	110.7
C1—Sn1—O3	90.9 (2)	C13—C17—H17B	110.7
C7—Sn1—C13	118.9 (2)	C16—C17—H17B	110.7
C7—Sn1—O1	90.6 (2)	H17A—C17—H17B	108.8
C7—Sn1—O3	87.8 (2)	O1—C18—O2	129.2 (6)
C13—Sn1—O1	82.8 (3)	O1—C18—C19	114.5 (5)
C13—Sn1—O3	96.5 (3)	O2—C18—C19	116.3 (5)
O1—Sn1—O3	177.7 (2)	F1—C19—F2	108.8 (5)
C18—O1—Sn1	119.3 (4)	F1—C19—C18	111.3 (5)
C20—O3—Sn1	117.0 (4)	F2—C19—C18	114.0 (5)
C29—N1—C22	113.5 (4)	F1—C19—Cl1	106.0 (4)
C29—N1—H1N1	108.9	F2—C19—Cl1	105.0 (5)
C29—N1—H1N1	108.9	C18—C19—Cl1	111.2 (5)
C22—N1—H1N2	108.9	O4—C20—O3	128.7 (6)
C22—N1—H1N2	108.9	O4—C20—C21	116.7 (5)
H1N1—N1—H1N2	107.7	O3—C20—C21	114.6 (6)
C2'—C1—C6'	121 (3)	F3—C21—F4	107.1 (5)
C2—C1—C6	115 (3)	F3—C21—C20	114.3 (5)
C2—C1—Sn1	123.3 (19)	F4—C21—C20	112.3 (5)
C2'—C1—Sn1	120.4 (18)	F3—C21—Cl2	103.7 (5)
C6'—C1—Sn1	118 (2)	F4—C21—Cl2	110.4 (5)
C6—C1—Sn1	122 (2)	C20—C21—Cl2	108.6 (4)
C3—C2—C1	125 (3)	N1—C22—C27	110.2 (5)
C3—C2—H2	117.5	N1—C22—C23	111.1 (5)
C1—C2—H2	117.5	C27—C22—C23	110.1 (5)
C4—C3—C2	116 (3)	N1—C22—H22	108.4
C4—C3—H3	122.1	C27—C22—H22	108.4
C2—C3—H3	122.1	C23—C22—H22	108.4
C3—C4—C5	124 (3)	C24—C23—C22	109.0 (6)
C3—C4—H4	117.8	C24—C23—H23A	109.9
C5—C4—H4	117.8	C22—C23—H23A	109.9
C4—C5—C6	116 (3)	C24—C23—H23B	109.9
C4—C5—H5	122.0	C22—C23—H23B	109.9
C6—C5—H5	122.0	H23A—C23—H23B	108.3
C5—C6—C1	124 (3)	C23—C24—C25	111.7 (5)
C5—C6—H6	118.0	C23—C24—H24A	109.3
C1—C6—H6	118.0	C25—C24—H24A	109.3
C3'—C2'—C1	118 (3)	C23—C24—H24B	109.3
C3'—C2'—H2'	120.9	C25—C24—H24B	109.3
C1—C2'—H2'	120.9	H24A—C24—H24B	107.9
C4'—C3'—C2'	122 (3)	C26—C25—C24	111.5 (5)
C4'—C3'—H3'	118.8	C26—C25—H25A	109.3
C2'—C3'—H3'	118.8	C24—C25—H25A	109.3
C3'—C4'—C5'	118 (3)	C26—C25—H25B	109.3
C3'—C4'—H4'	121.2	C24—C25—H25B	109.3
C5'—C4'—H4'	121.2	H25A—C25—H25B	108.0
C4'—C5'—C6'	123 (3)	C25—C26—C27	112.1 (5)
C4'—C5'—H5'	118.7	C25—C26—H26A	109.2
C6'—C5'—H5'	118.7	C27—C26—H26A	109.2
C5'—C6'—C1	118 (3)	C25—C26—H26B	109.2
C5'—C6'—H6'	121.2	C27—C26—H26B	109.2
C1—C6'—H6'	121.2	H26A—C26—H26B	107.9
C8—C7—C12	118.7 (5)	C22—C27—C26	109.6 (5)
C8—C7—Sn1	119.9 (4)	C22—C27—H27A	109.8
C12—C7—Sn1	121.2 (4)	C26—C27—H27A	109.8
C7—C8—C9	119.3 (6)	C22—C27—H27B	109.8
C7—C8—H8	120.4	C26—C27—H27B	109.8
C9—C8—H8	120.4	H27A—C27—H27B	108.2
C10—C9—C8	120.8 (6)	N1—C29—C30	111.2 (5)
C10—C9—H9	119.6	N1—C29—C34	110.6 (5)
C8—C9—H9	119.6	C30—C29—C34	111.2 (5)
C11—C10—C9	120.6 (6)	N1—C29—H29	107.9
C11—C10—H10	119.7	C30—C29—H29	107.9
C9—C10—H10	119.7	C34—C29—H29	107.9
C10—C11—C12	119.7 (6)	C29—C30—C31	109.5 (6)
C10—C11—H11	120.1	C29—C30—H30A	109.8
C12—C11—H11	120.1	C31—C30—H30A	109.8
C7—C12—C11	120.9 (6)	C29—C30—H30B	109.8
C7—C12—H12	119.5	C31—C30—H30B	109.8
C11—C12—H12	119.5	H30A—C30—H30B	108.2
C14—C13—C17	105.1 (5)	C32—C31—C30	112.7 (7)
C14—C13—Sn1	123.1 (5)	C32—C31—H31A	109.1
C17—C13—Sn1	114.6 (4)	C30—C31—H31A	109.1
C14—C13—H13	104.0	C32—C31—H31B	109.1
C17—C13—H13	104.0	C30—C31—H31B	109.1
Sn1—C13—H13	104.0	H31A—C31—H31B	107.8
C13—C14—C15	105.2 (5)	C31—C32—C33	111.1 (6)
C13—C14—H14A	110.7	C31—C32—H32A	109.4
C15—C14—H14A	110.7	C33—C32—H32A	109.4
C13—C14—H14B	110.7	C31—C32—H32B	109.4
C15—C14—H14B	110.7	C33—C32—H32B	109.4
H14A—C14—H14B	108.8	H32A—C32—H32B	108.0
C16—C15—C14	105.8 (4)	C32—C33—C34	111.3 (6)
C16—C15—H15A	110.6	C32—C33—H33A	109.4
C14—C15—H15A	110.6	C34—C33—H33A	109.4
C16—C15—H15B	110.6	C32—C33—H33B	109.4
C14—C15—H15B	110.6	C34—C33—H33B	109.4
H15A—C15—H15B	108.7	H33A—C33—H33B	108.0
C15—C16—C17	107.4 (4)	C29—C34—C33	110.9 (6)
C15—C16—H16A	110.2	C29—C34—H34A	109.5
C17—C16—H16A	110.2	C33—C34—H34A	109.5
C15—C16—H16B	110.2	C29—C34—H34B	109.5
C17—C16—H16B	110.2	C33—C34—H34B	109.5
H16A—C16—H16B	108.5	H34A—C34—H34B	108.1
			
C13—Sn1—O1—C18	169.9 (5)	C8—C9—C10—C11	−1.3 (10)
C7—Sn1—O1—C18	50.8 (5)	C9—C10—C11—C12	0.6 (10)
C1—Sn1—O1—C18	−68.4 (5)	C8—C7—C12—C11	−1.0 (9)
C13—Sn1—O3—C20	60.0 (5)	Sn1—C7—C12—C11	173.6 (4)
C7—Sn1—O3—C20	178.9 (5)	C10—C11—C12—C7	0.6 (9)
C1—Sn1—O3—C20	−62.0 (5)	C7—Sn1—C13—C14	−61.9 (8)
C13—Sn1—C1—C2	−108.5 (13)	C1—Sn1—C13—C14	124.4 (6)
C7—Sn1—C1—C2	77.8 (13)	O3—Sn1—C13—C14	29.2 (7)
O3—Sn1—C1—C2	−10.2 (13)	O1—Sn1—C13—C14	−148.5 (7)
O1—Sn1—C1—C2	169.2 (13)	C7—Sn1—C13—C17	168.2 (5)
C13—Sn1—C1—C2'	−121.7 (13)	C1—Sn1—C13—C17	−5.5 (7)
C7—Sn1—C1—C2'	64.6 (13)	O3—Sn1—C13—C17	−100.6 (6)
O3—Sn1—C1—C2'	−23.5 (13)	O1—Sn1—C13—C17	81.6 (6)
O1—Sn1—C1—C2'	155.9 (13)	C17—C13—C14—C15	−32.5 (8)
C13—Sn1—C1—C6'	56.8 (11)	Sn1—C13—C14—C15	−166.2 (6)
C7—Sn1—C1—C6'	−116.9 (11)	C13—C14—C15—C16	19.2 (9)
O3—Sn1—C1—C6'	155.1 (11)	C14—C15—C16—C17	1.1 (9)
O1—Sn1—C1—C6'	−25.5 (11)	C14—C13—C17—C16	33.2 (8)
C13—Sn1—C1—C6	70.4 (11)	Sn1—C13—C17—C16	171.5 (5)
C7—Sn1—C1—C6	−103.4 (11)	C15—C16—C17—C13	−21.0 (8)
O3—Sn1—C1—C6	168.6 (11)	Sn1—O1—C18—O2	6.9 (9)
O1—Sn1—C1—C6	−12.0 (11)	Sn1—O1—C18—C19	−173.6 (4)
C2'—C1—C2—C3	−102 (18)	O1—C18—C19—F1	−56.4 (7)
C6'—C1—C2—C3	13.2 (19)	O2—C18—C19—F1	123.1 (6)
C6—C1—C2—C3	−0.2 (3)	O1—C18—C19—F2	180.0 (5)
Sn1—C1—C2—C3	178.7 (11)	O2—C18—C19—F2	−0.5 (8)
C1—C2—C3—C4	0.0 (3)	O1—C18—C19—Cl1	61.5 (6)
C2—C3—C4—C5	0.0 (7)	O2—C18—C19—Cl1	−118.9 (5)
C3—C4—C5—C6	0.2 (9)	Sn1—O3—C20—O4	1.0 (9)
C4—C5—C6—C1	−0.5 (9)	Sn1—O3—C20—C21	179.9 (4)
C2—C1—C6—C5	0.5 (7)	O4—C20—C21—F3	4.9 (8)
C2'—C1—C6—C5	13 (2)	O3—C20—C21—F3	−174.1 (6)
C6'—C1—C6—C5	−102 (19)	O4—C20—C21—F4	127.2 (6)
Sn1—C1—C6—C5	−178.4 (12)	O3—C20—C21—F4	−51.8 (7)
C2—C1—C2'—C3'	70 (18)	O4—C20—C21—Cl2	−110.4 (6)
C6'—C1—C2'—C3'	−0.2 (3)	O3—C20—C21—Cl2	70.6 (6)
C6—C1—C2'—C3'	−13 (2)	C29—N1—C22—C27	76.9 (6)
Sn1—C1—C2'—C3'	178.3 (11)	C29—N1—C22—C23	−160.8 (5)
C1—C2'—C3'—C4'	0.0 (3)	N1—C22—C23—C24	177.0 (5)
C2'—C3'—C4'—C5'	0.0 (6)	C27—C22—C23—C24	−60.6 (7)
C3'—C4'—C5'—C6'	0.2 (8)	C22—C23—C24—C25	57.4 (8)
C4'—C5'—C6'—C1	−0.5 (8)	C23—C24—C25—C26	−54.2 (9)
C2—C1—C6'—C5'	−12 (3)	C24—C25—C26—C27	53.0 (7)
C2'—C1—C6'—C5'	0.5 (6)	N1—C22—C27—C26	−177.6 (5)
C6—C1—C6'—C5'	72 (18)	C23—C22—C27—C26	59.5 (7)
Sn1—C1—C6'—C5'	−178.1 (11)	C25—C26—C27—C22	−56.0 (7)
C13—Sn1—C7—C8	−46.3 (6)	C22—N1—C29—C30	−151.5 (5)
C1—Sn1—C7—C8	127.6 (4)	C22—N1—C29—C34	84.5 (6)
O3—Sn1—C7—C8	−142.5 (5)	N1—C29—C30—C31	179.5 (6)
O1—Sn1—C7—C8	35.8 (5)	C34—C29—C30—C31	−56.8 (7)
C13—Sn1—C7—C12	139.2 (5)	C29—C30—C31—C32	55.6 (8)
C1—Sn1—C7—C12	−46.9 (5)	C30—C31—C32—C33	−54.3 (8)
O3—Sn1—C7—C12	42.9 (4)	C31—C32—C33—C34	54.0 (9)
O1—Sn1—C7—C12	−138.8 (4)	N1—C29—C34—C33	−177.8 (5)
C12—C7—C8—C9	0.3 (9)	C30—C29—C34—C33	58.1 (8)
Sn1—C7—C8—C9	−174.4 (5)	C32—C33—C34—C29	−55.9 (8)
C7—C8—C9—C10	0.9 (10)		

### Hydrogen-bond geometry (Å, °)

**Table:**

*D*—H···*A*	*D*—H	H···*A*	*D*···*A*	*D*—H···*A*
N1—H1N1···O2	0.88	1.88	2.758 (6)	173
N1—H1N2···O4^i^	0.88	1.93	2.804 (6)	169

Symmetry codes: (i) −*x*+1, *y*+1/2, −*z*+1.
